# Collagen Dynamics During the Process of Osteocyte Embedding and Mineralization

**DOI:** 10.3389/fcell.2019.00178

**Published:** 2019-09-18

**Authors:** Lora A. Shiflett, LeAnn M. Tiede-Lewis, Yixia Xie, Yongbo Lu, Eleanor C. Ray, Sarah L. Dallas

**Affiliations:** ^1^Department of Oral and Craniofacial Sciences, School of Dentistry, University of Missouri-Kansas City, Kansas City, MO, United States; ^2^Department of Biomedical Sciences, Texas A&M University College of Dentistry, Dallas, TX, United States

**Keywords:** osteocytes, collagen, extracellular matrix, live cell imaging, motility, bone mineralization, embedding

## Abstract

Bone formation, remodeling and repair are dynamic processes, involving cell migration, ECM assembly, osteocyte embedding, and bone resorption. Using live-cell imaging, we previously showed that osteoblast assembly of the ECM proteins fibronectin and collagen is highly dynamic and is integrated with cell motility. Additionally, osteoblast-to-osteocyte transition involved arrest of cell motility, followed by dendrite extension and retraction that may regulate positioning of embedding osteocytes. To further understand how osteocytes differentiate and embed in collagen, mice were generated that co-expressed GFP*topaz*-tagged collagen with a Dmp1-Cre-inducible tdTomato reporter targeted to preosteocytes/osteocytes. Dual live-cell imaging of collagen and osteocyte dynamics in mineralizing primary calvarial cell cultures showed that Dmp1-Cre/tdTomato turned on in early bone nodule forming regions, demarcated by foci of concentrated GFP-collagen bundles that appeared structurally distinct from the surrounding collagen. Dmp1-Cre/tdTomato-positive cells were post-mitotic and were continuously induced throughout the 2 week timecourse, whereas the majority of collagen was assembled by day 7. GFP-collagen fibrils showed global (tissue-level) motions, suggesting coordinated cell layer movement, and local fibril motions mediated by cell-generated forces. Condensation of collagen fibril networks occurred within bone nodules prior to mineralization. Intravital imaging confirmed a similar structural appearance of GFP-collagen in calvarial bone, with analogous global motions of mineralizing areas adjacent to sutures. In early (unmineralized) calvarial cell cultures, Dmp1-Cre/tdTomato-positive cells were motile (mean velocity 4.8 μm/h), moving freely in and around the forming bone nodule, with a small number of these cells embedded in collagen, constraining their motion. In mineralizing cultures, the average velocity of Dmp1-Cre/tdTomato-positive cells was significantly reduced (0.7 μm/h), with many immobilized in the mineralizing nodule. Three apparent mechanisms for embedding of Dmp1-Cre/tdTomato-positive cells were observed. In some cases, a previously motile Dmp1-Cre/tdTomato-positive cell became immobilized in collagen fibril networks that were newly assembled around the cell, thereby entrapping it. In other cases, a motile Dmp1-Cre/tdTomato-positive cell moved into an already formed “collagen lacuna,” arrested its motility and became embedded. Alternatively, some cells switched on tdTomato expression *in situ* within a lacuna. These data provide new insight into the dynamic process of bone collagen assembly and suggest multiple mechanisms for osteocyte entrapment in collagen matrix.

## Introduction

Our understanding of mineralized tissue biology has come predominantly from static imaging approaches, such as histology/histomorphometry, light and electron microscopy and micro computed tomography, combined with chemical, biochemical, protein and molecular biological analyses, together with manipulation of mouse genetics (Halloran et al., 2002; Faibish et al., 2005; Mckee et al., 2005; Murshed et al., 2005; Huitema and Vaandrager, 2007; Pacureanu et al., 2012). These approaches have led to an in depth understanding of many of the molecular pathways controlling development and postnatal growth of the skeleton as well as skeletal aging and various bone pathologies. However, the biological processes that occur in bone tissues, such as bone formation, remodeling and repair are highly coordinated dynamic processes, involving cell migration and embedding, extracellular matrix (ECM) assembly, and bone resorption. Static imaging approaches do not convey the dynamic nature of these events and may therefore fall short in providing a comprehensive understanding of the underlying biology. In contrast, live cell imaging allows us to visualize active processes in living cells, organs, whole embryos and whole animals from a temporospatial perspective and enables cellular, subcellular and tissue behavior to be quantified as a function of time. Live cell imaging is increasingly being used in various model systems to obtain quantitative insight into intracellular and extracellular processes, such as organelle transport, mitochondrial function, autophagy and energy metabolism, assembly and reorganization of the ECM as well as to study embryonic development/morphogenesis and stem cell function (Eils and Athale, 2003; Friedl, 2004; Kulesa, 2004; Dallas et al., 2006; Sivakumar et al., 2006; Zamir et al., 2008; Lo Celso et al., 2009; Tiede et al., 2009; Xie et al., 2009; Appelhans and Busch, 2017; Bell, 2017; Bigley et al., 2017; Ratnayake and Currie, 2017; Lu et al., 2018).

Important insights into the dynamic process of extracellular matrix assembly and organization have come from live cell imaging of ECM proteins, such as fibronectin, collagen, elastin, fibrillins and latent transforming growth factor beta binding proteins (LTBPs) (Czirok et al., 2004; Davidson et al., 2004; Filla et al., 2004; Dallas et al., 2006; Kozel et al., 2006; Sivakumar et al., 2006; Aleksandrova et al., 2012; Lu et al., 2018). A common theme emerging from these studies is that assembly of many of these ECM proteins is highly dynamic and appears to be integrated with cell motility. These cell motions exert forces on the fibrils, which stretch and distort them as they are being assembled. Our laboratory has recently developed GFP*topaz-* and mCherry-tagged type I collagen fusion protein constructs and stably transfected them into MLO-A5 osteoblast-like cells and fibronectin-null mouse embryonic fibroblasts (Lu et al., 2018). Live cell imaging using these cell models revealed the dynamic nature of type I collagen assembly and showed its dependence on fibronectin assembly (Lu et al., 2018). A particularly interesting observation from these studies was that osteoblasts were able to physically reshape the collagen fibrillar network by pushing collagen outwards to form hole-like structures. We hypothesized that this reshaping of the collagen ECM to form holes in the network may provide a mechanism for formation of a nascent osteocyte lacuna in bone.

Osteocytes make up over 90% of the cells in bone, but because they are embedded within a mineralized matrix, they have been challenging to study. These terminally differentiated cells are derived from osteoblasts that become embedded within the ECM they produce, termed osteoid, which then becomes mineralized (reviewed in Dallas et al., 2013; Jilka and O’brien, 2016; Prideaux et al., 2016). The transition from osteoblast to osteocyte involves a dramatic change in morphology from a polygonal cell to a cell with a reduced cytoplasmic volume and a highly dendritic morphology, reminiscent of neuronal cells. Differentiation from osteoblast to osteocyte is associated with downregulation of osteoblast expressed genes, such as type I collagen (*COL1A1* and *COL1A2*), alkaline phosphatase (*TNSALP*), and RUNX family transcription factor 2 (*RUNX2*), concomitant with upregulation of osteocyte marker genes including E11/gp38/podoplanin (*PDPN*), dentin matrix protein 1 (*DMP1*), matrix extracellular phosphoglycoprotein (*MEPE*), phosphate-regulating gene with homologies to endopeptidases on the X chromosome (*PHEX*), and fibroblast growth factor 23 (*FGF23*) (Schulze et al., 1999; Toyosawa et al., 2001; Igarashi et al., 2002; Franz-Odendaal et al., 2006; Ubaidus et al., 2009; reviewed in Dallas et al., 2013). Differentiation to the mature osteocyte phenotype is associated with expression of the *SOST* gene, which encodes the protein, sclerostin (Winkler et al., 2003).

Various mechanisms have been proposed to explain how osteoblasts embed to become osteocytes. One theory proposes that embedding is a passive process in which osteoblasts slow down their production of extracellular matrix and then become “buried alive” in the osteoid produced by neighboring osteoblasts (Palumbo et al., 1990; Nefussi et al., 1991; Franz-Odendaal et al., 2006). However, other researchers have proposed that osteocyte embedding is an active, invasive process, involving proteolytic degradation of the extracellular matrix to form the osteocyte lacuna and canaliculi (Zhao et al., 2000; Holmbeck et al., 2005). To further understand the dynamic mechanisms by which osteocytes differentiate and embed in collagen, this study set out to perform dual imaging of osteocyte differentiation using a lineage reporter, together with imaging collagen using a fluorescently tagged collagen fusion protein. To accomplish this, transgenic mice were generated that co-expressed a GFP*topaz*-tagged type I collagen fusion protein, together with a Dmp1-Cre-inducible tdTomato reporter targeted to preosteocytes/osteocytes. Dual live cell imaging of collagen fibril assembly dynamics and osteocyte differentiation and embedding was performed in mineralizing primary bone cells isolated from these mice and intravital imaging was also performed in these mice. The findings reveal the dynamic nature of collagen assembly by mineralizing bone cells and suggest multiple mechanisms for osteocyte entrapment in collagen matrix.

## Materials and Methods

### Reagents

Unless stated otherwise, standard reagents and chemicals were from Sigma Aldrich (St. Louis, MO, United States), or Thermo Fisher Scientific (Waltham, MA, United States). Rat tail collagen was from Becton-Dickinson (Franklin Lakes, NJ, United States). L-ascorbic acid phosphate magnesium salt n-hydrate was from Wako Chemicals USA, Inc., Richmond, VA, United States.

### Animals

The Ai9 mouse, hereafter referred to as tdTomato, was from the Jackson Laboratory, Bar Harbor, ME, United States [B6.Cg-Gt(ROSA)26Sor^TM 9(CAG–tdTomato)Hze^, JAX Stock #007909] and is a Cre reporter mouse with a STOP cassette flanked by LoxP sites (Madisen et al., 2010). The STOP cassette blocks transcription of the tdTomato red fluorescent protein variant and tdTomato fluorescence is induced following Cre-mediated recombination. The 10 kb Dmp1-Cre mouse was kindly provided by Dr. Jian Q. Feng (Texas A&M College of Dentistry) and has been described previously (Lu et al., 2007). GFP-collagen transgenic mice were generated in our laboratory by inserting a GFP*topaz* tag into the mouse proα2(I) collagen N-terminus under control of the 3.6 kb type I collagen promoter (Kamel-Elsayed et al., 2015 and manuscript in preparation). These transgenic mice were generated on a C57BL/6N background by pronuclear injection at the Transgenic Technology Center at the University of Texas Southwestern Medical Center, Dallas, TX, United States. Mice were bred to generate GFP-col^+ ⁣/−^/Dmp1-Cre^+ ⁣/−^/tdTomato^+ ⁣/−^ mice, which have green fluorescent collagen and a red fluorescent lineage reporter for preosteocytes/osteocytes. The mice were genotyped by PCR of tail DNA samples. For tdTomato mice, PCR was performed according to the Jackson Laboratory protocol. Genotyping of Dmp1-Cre transgenic mice was performed using forward primer, 5′-CCAAGCCCTGAAAATCACAGA-3′ and reverse primer, 5′-CCTGGCGATCCCTGAACATG-3′. Genotyping of GFP-collagen transgenic mice was performed using forward primer 5′-TCATCTGCACCACCGGCAAGC-3′ and reverse primer 5′-AGCAGGACCATGTGATCGCGC-3′. Expression of the fluorescent transgenes was confirmed by examining tail clip biopsies under a Nikon TE300 widefield epifluorescence microscope. Animal experiments and euthanasia were performed under an approved IACUC protocol at the University of Missouri Kansas City (UMKC), and conformed to relevant federal guidelines. The UMKC animal facility is AAALAC approved and animal care and husbandry meets requirements in the Guide for the Care and use of Laboratory Animals (8th Ed.), National Research Council. Animals were group housed on a 12 h light/dark cycle with *ad libitum* food and water at 22°C constant temperature and 45–55% humidity.

### Fluorescent Bone Histology

Femurs from 8 week old GFP-collagen transgenic mice or Dmp1-Cre^+ ⁣/−^/tdTomato^+ ⁣/−^ mice were prepared for cryosectioning as described previously (Kamel-Elsayed et al., 2015). Briefly, after fixation in 4% paraformaldehyde in PBS, the femurs were decalcified in 10% EDTA, pH 7.4 for 1–2 weeks, then equilibrated in 15% then 30% sucrose in PBS before embedding and freezing in Tissue-Tek O.C.T compound (Sakura Finetek USA Inc., Torrence, CA, United States). 6μm longitudinal sections were cut on a Leica CM3050S cryomicrotome (Leica Microsystems, Wetzlar, Germany). Slides were either viewed without staining or the nuclei were stained using a 5 min incubation in 4 μg/ml 4′-6-Diamidino-2-phenylindole (DAPI) (Thermo Fisher Scientific, Waltham, MA, United States) in PBS. Sections were coverslip mounted in 1:1 glycerol:PBS with 1 mM MgCl_2__._ Sections were imaged using a 20x 0.75 NA or 10x 0.45 NA objective on a Nikon E800 widefield epifluorescence microscope (Nikon Instruments Inc., Mellville, NY, United States) with filter sets optimized for each fluorophore. Digital photographs were taken using an Optronics CCD color camera (Optronics, Goleta, CA, United States) interfaced with the AnalySIS software (Soft Imaging System GmbH, Muenster, Germany).

### Cell Culture

Unless stated otherwise tissue culture reagents were from Gibco (Life Technologies Inc., Grand Island, NY, United States), GE Healthcare Life Sciences (Marlborough, MA, United States), or Mediatech Inc. (Herndon, VA, United States). Heat inactivated fetal bovine serum (FBS) was from Hyclone Laboratories (GE Healthcare Life Sciences) or Atlanta Biologicals (Flowery Branch, GA, United States). Primary calvarial cells were routinely maintained in growth medium consisting of α-Minimum Essential Medium (α-MEM) containing 10% heat inactivated FBS, 2 mM L-glutamine (LG) and 100 U/ml penicillin/streptomycin (P/S) in a humidified 5% CO_2_ incubator at 37°C. The preparation of these primary cells is described below. For experiments, the cells were plated onto rat tail collagen-coated Lab-Tek coverglass chamber slides (Thermo Fisher Scientific, Waltham, MA, United States) at 4 × 10^4^ cells/cm^2^ growth area in growth medium. At confluence (day 0), the medium was changed to osteogenic medium consisting of α-MEM supplemented with 5% FBS, 100 U/ml P/S, 2 mM LG, 5 mM β-Glycerophosphate (βGP) and 50 μg/ml ascorbic acid, to promote osteogenic differentiation. In some experiments 0.5 mM βGP was used for days 0–5 and then 5 mM βGP was used from day 5 onwards. The cells were cultured for up to 14–21 days, with media refreshed every 3 days. Imaging was initiated at various times as indicated in the results section. During imaging 100 μg/ml L-ascorbic acid phosphate magnesium salt n-hydrate was used in place of standard ascorbic acid, since it is more stable under imaging conditions.

### Primary Calvarial Cell Isolation

Primary mouse calvarial cells were isolated from 7 to 8 day old neonatal mouse calvaria by sequential trypsin and collagenase digestions using a modification of methods described previously for fetal rats (Harris et al., 1994; Dallas et al., 1995). Briefly, the parietal and frontal bones were aseptically dissected and subjected to four 20 min sequential digests in 0.2% collagenase/0.05% trypsin in α-MEM (no additives) on a shaker plate at 37°C. Digests 2–4 were kept as the osteoblast-enriched cell population and first or second passage cells were plated for experiments.

### Live Cell Imaging

For live cell imaging, cells were plated onto collagen-coated Lab-Tek coverglass chamber slides (Thermo Fisher Scientific, Waltham, MA, United States) and differentiated as described above. On the day of imaging, the media was refreshed with additives as above, but with 100 μg/ml L-ascorbic acid phosphate magnesium salt n-hydrate (a more stable form of ascorbic acid). Live cell imaging was performed on an automated Nikon TE 2000E widefield epifluorescence microscope with precision motorized x, y, and z stage using a 20x 0.75 NA objective with filter sets optimized for each fluorophore. The “Metamorph” software (Molecular Devices, LLC, Sunnyvale, CA, United States) controlled the microscope and multidimensional imaging parameters. Temperature was held constant at 37°C using a microscope incubator cabinet (“The Box” with “The Cube,” Life Imaging Systems, Reinach, Switzerland) and a humidified 5% CO_2_ atmosphere was maintained using a gas mixer (“The Brick,” Life Imaging Systems) in conjunction with a humidified stage top incubation chamber. Images were acquired for each time point under epifluorescent illumination using a Photometrics Coolsnap HQ cooled CCD camera with 12-bit gray scale resolution. Fields of 450 × 335μm were imaged at a spatial resolution of 696 × 520 pixels (with 2 × 2 binning) every 30 min for up to 14 days from 5 optical planes, with media refreshed every 48 h. Image stacks were processed in Metamorph using the “best focus” algorithm or by manually selecting the best focus plane and then exported as image stack (.stk) files. Post-acquisition image processing including contrast adjustments, color merging, conversion to 8-bit or RGB stacks, stack processing and compilation into movies was done using ImageJ software. Image stacks were registered using the “stackreg” plugin in ImageJ (translation mode) (Thevenaz et al., 1998) to align the time series stacks and correct for misaligned image frames. Aligned image stacks were assembled into movies using ImageJ. Gamma adjustments were applied to some of the collagen-GFP movies to allow visualization of collagen fibrils at early time points due to the large dynamic range of fluorescence intensity between early and late time points and to some of the Dmp1-Cre/tdTomato movies to allow better visualization of low intensity pixels at the cell boundaries. These are indicated in the legends for the Supplementary Movies.

### Intravital Imaging

Intravital imaging was performed on 2 week old mice. The mice were anesthetized with 75 mg/Kg ketamine and 0.5 mg/Kg dexmedetomidine (Henry Schein Animal Health, Dublin, OH). Once under a deep plane of anesthesia, the surgical site was prepared by shaving the fur on the top of the head with an electric clipper and cleaning with betadyne followed by 70% ethanol (repeated three times). The skin overlying the calvarium was then aseptically dissected using iris scissors and reflected to expose the surface of the calvarium (mainly the parietal and frontal bones). Loose adherent connective tissue was gently dissected from the bone surface with sterile forceps under a dissection microscope. The mouse was then immobilized using a custom stereotaxic holder designed for the inverted confocal microscope platform, with the mouse lain ventral side upwards and the exposed calvarial surface apposed to a sterile glass coverslip window, allowing imaging from below. To maintain hydration of the exposed calvarial surface, an imaging gel consisting of 300 mM D-sorbitol and 0.5% carbomer940 pH 7.4 (Rothstein et al., 2006) was placed between the exposed calvarial surface and the glass coverslip. To ensure that deep anesthesia was maintained, the animals were monitored throughout the imaging period by assessment of toe pinch reflex, respiratory rate, and checking for spontaneous movement or other signs of discomfort. Deep anesthesia was maintained by administering an additional half dose of ketamine/dexmedetomidine if needed. Sterile saline was injected subcutaneously prior to surgery to maintain hydration (500 μl/10 g body weight). To prevent hypothermia, the air temperature in the microscope incubation cabinet was held at 30°C. At the end of the imaging period, while still under deep anesthesia, the animals were humanely euthanized.

Intravital imaging was performed using a Leica TCS Sp5 II scanning confocal microscope in resonant scanner mode interfaced with the LAS-AF software (Leica Microsystems, Wetzlar, Germany). Images were acquired at a spatial resolution of 1024 × 1024 pixels using a 20x 0.7 NA objective with 4x digital zoom, a pinhole size of 1.2 AU and using line averaging of 48. Images were acquired for GFP-collagen and tdTomato fluorescence from 7 optical planes with a step size of 2 μm. The 488 nm laser (15% output) was used for excitation of GFP-collagen and the 543nm laser (20% output) was used for excitation of tdTomato. Collection windows were optimized for each fluorophore using the Leica SP prism and tunable slider system and signal was collected using HyD detectors. Post-acquisition image processing was done using ImageJ software, as described above.

### Cell Tracking and Quantitation of Collagen-GFP and tdTomato Assembly Dynamics

For tracking of cell velocities and cell motions, registered image stacks were used and the motion trajectories for individual cells within each movie field were plotted using the MTrackJ Plugin in ImageJ (Meijering et al., 2012). As an additional analysis, cells that had their motion trajectories plotted were scored according to whether they arose by directly switching on tdTomato expression or via mitotic division of a cell expressing tdTomato (note: cells that were already tdTomato-positive at the start of the movies were excluded from this scoring analysis). To quantify collagen-GFP and tdTomato fluorescence over time in long-term timelapse movies, background subtracted movie stacks were thresholded in ImageJ and the collagen fibril area or tdTomato fluorescence area was quantified from thresholded stacks using the Analyze Particles Plugin in ImageJ (Meijering et al., 2012).

## Results

### Expression of Fluorescent Transgenes in GFP-Collagen/Dmp1-Cre/tdTomato Mice

**FIGURE 1:**
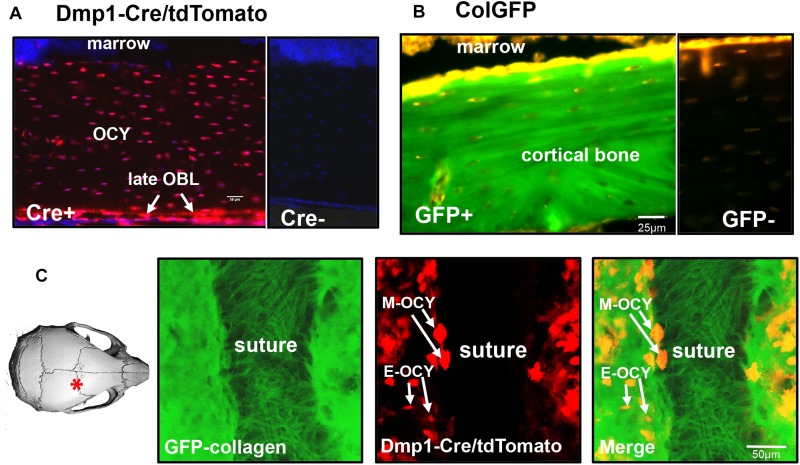
Expression of fluorescent transgenes in GFP-collagen and Dmp1-Cre/tdTomato mice. **(A)** tdTomato expression in the femur (longitudinal section) of an 8 week old Dmp1Cre/tdTomato transgenic mouse (Cre+). A Cre negative littermate control is shown at right for comparison (Cre-). tdTomato expression is shown in red and DAPI nuclear stain is shown in blue. (OCY, osteocytes; late OBL, late osteoblasts), bar = 50 μm. **(B)** GFP-collagen expression in the femur (longitudinal section) an 8 week old GFP-collagen transgenic mouse (GFP+). A GFP-negative littermate is shown at right for comparison (GFP-). GFP-collagen is shown in green and tissue autofluorescence appears yellow/orange, bar = 25 μm. **(C)** Still frame from intravital imaging in a 15 day old GFP-collagen/Dmp1-Cre/tdTomato transgenic mouse. The image on the left illustrates the location of the imaging field. GFP collagen is shown in green and tdTomato is shown in red. (E-OCY, embedded osteocytes; M-OCY, larger tdTomato-positive cells that are motile, located at the edge of the suture), bar = 50 μm. Panel **(A)** is modified from Dallas et al. (2018) with permission.

Figure 1A shows expression of the tdTomato transgene in the femur of Dmp1-Cre^+ ⁣/−^/tdTomato^+ ⁣/−^ transgenic mice. In this model, the tdTomato reporter is expressed following Cre-mediated recombination driven by the 10 kb *Dmp1* promoter. This promoter was originally thought to be expressed in preosteocytes, osteocytes, odontoblasts and some pulp cells (Toyosawa et al., 2001; Feng et al., 2003; Kalajzic et al., 2004; Lu et al., 2007). However, subsequent studies have shown that it is expressed in some late osteoblasts as well (Kalajzic et al., 2013; Dallas et al., 2018), which may be due to the sensitivity of the tdTomato reporter in the Ai9 mouse line, even to extremely low levels of Cre recombinase (reviewed in Dallas et al., 2018). In our hands, in bone tissue, the tdTomato transgene was strongly expressed in the majority of embedded osteocytes, preosteocytes, and some late osteoblasts on the bone surface when used with the 10 kb Dmp1 Cre driver (Figure 1A) (a Cre negative littermate control is shown at right). Figure 1B shows the localization of GFP-tagged collagen in the femur of a GFP-collagen transgenic mouse. Bright GFP-collagen fluorescence was observed in the extracellular matrix of the bone (a GFP negative littermate control is shown at right). Figure 1C shows still frames from intravital imaging in a GFP-collagen^+ ⁣/−^/Dmp1-Cre^+ ⁣/−^/tdTomato^+ ⁣/−^ triple transgenic mouse to illustrate the expression of GFP-collagen and tdTomato at the suture region of the parietal bone (see left image of a mouse skull for the location of the imaged region). GFP-collagen fiber bundles could be clearly visualized in the suture area, with a much more densely packed appearance of the GFP-collagen in the mineralized bone regions on either side of the suture. Many tdTomato-positive cells were found embedded in lacunae in the GFP-collagen bone matrix (E-OCY). Additionally, larger tdTomato-positive cells were observed on the bone at the edges of the suture and on the bone surface (M-OCY), presumably representing cells that retain properties of osteoblasts, but are transitioning toward becoming future osteocytes. These cells were also found to be motile (data not shown). tdTomato-positive cells were not observed in the suture region.

### Dynamics of Collagen Assembly in Mineralizing Primary Calvarial Cell Cultures

**FIGURE 2:**
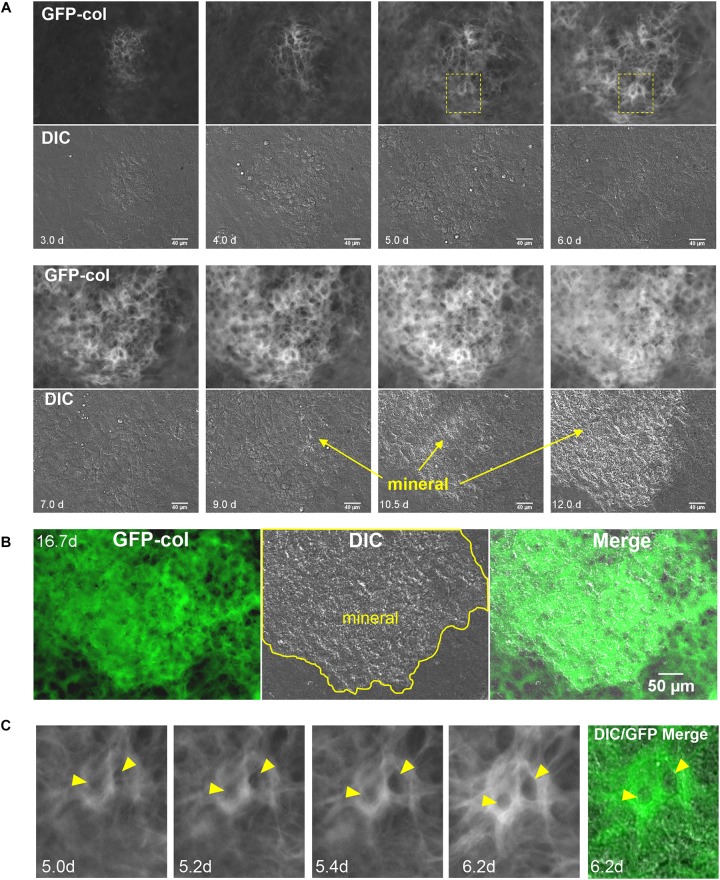
Live cell imaging of collagen assembly dynamics in long-term osteogenic calvarial cell cultures. **(A)** Still frames from timelapse imaging showing formation of a mineralized bone nodule from its initial condensation at day 3 to mineralization by day 12. GFP-collagen is shown in the upper panels and DIC images are shown in the lower panels. Mineral deposition can be seen on the DIC images and is initiated around day 9 (bar = 40 μm). To appreciate the dynamic nature of this collagen assembly please view Supplementary Movies 1–4. **(B)** Still frames from day 16.7 in the same timelapse series showing GFP-collagen, DIC and merged images. Note that the mineral is deposited exclusively in the collagen-rich focal area (bar = 50 μm). **(C)** Still frames showing an enlargement of the boxed area in (A), illustrating GFP-collagen dynamics during the formation of two osteocyte lacunae (arrowheads) within the bone matrix over days 5.0–6.2. A merged GFP-collagen/DIC image is shown at right for the 6.2 h time point. To view this dynamically, please see Supplementary Movie 3, arrowheads.

Having established that the GFP-collagen and Dmp1-Cre/tdTomato transgenes showed the expected localizations in bone, primary calvarial cells were isolated from these transgenic mice and differentiated *in vitro* to form mineralized bone nodules using osteogenic media containing ascorbic acid and β-GP. This culture system was used as an *in vitro* model to determine the dynamics of collagen assembly as well as the dynamic process by which osteocytes become embedded in bone collagen matrix. Long-term timelapse imaging was performed for up to 14 days. First, the GFP-collagen movies were examined, which allowed visualization of collagen assembly over time as the primary calvarial cells formed foci that progressed to form mineralized bone nodules. Figures 2A,B show a series of still frames from live imaging of a developing bone nodule starting at 3 days of culture in osteogenic media and continuing to 16.7 days. Supplementary Movie 1 shows the entire movie sequence from the initial condensation of cells at around 3 days to form an early nodule through expansion and maturation of the nodule and its mineralization. Since this movie represents a full 2 weeks of live cell imaging, we have also divided the movie into shorter segments to better illustrate the dynamic events at different stages throughout the differentiation and mineralization process.

Supplementary Movie 2 shows the time period from d3 to d4, representing the early stages of differentiation (also see still frames in Figure 2A). At these early stages, the initial formation of a future bone nodule is seen in the DIC images as a condensation of cells in a focal area where the cells became rounded/multilayered. GFP-collagen was deposited throughout the cell layer, but was brighter and more concentrated in these foci of condensed cells that would become future bone nodules. Many of these GFP-collagen containing foci underwent expansion/stretching whereby the collagen was pushed outwards from the center of the forming nodule, as shown in Supplementary Movie 2. This expansion appeared to be mediated by coordinated movement of cells outwards from the center of the nodule and further condensation of cells to increase the nodule size. In addition, there was considerable assembly of new collagen in both the forming nodule and the adjacent cell layer. The cells participating in nodule formation appeared more rounded and showed extensive membrane ruffling (visible in the movies as bright hair-like features on the cells) compared to the cells in the surrounding monolayer. Additionally, the cells in both the forming nodule and surrounding monolayer were constantly in motion, which exerted forces on the forming fibrils, resulting in continual stretching/small deformations of the fibrils during the assembly and expansion process.

Supplementary Movie 3 depicts the time period from d4 to d8 (also see still frames in Figure 2A). Over this period, the foci continued to expand outwards and assembled brighter and thicker GFP-collagen fibril networks. As the collagen fiber network matured, the collagen in the nodule forming foci appeared structurally distinct from the surrounding collagen and by day 7 had a honeycomb-like appearance, with well-defined “holes” in the collagen network that probably represent future osteocyte lacunae. These presumptive lacunae appeared to be initially formed by cells pushing collagen fibrils outwards to form a hole, followed by new assembly of collagen fibrils around the periphery and compaction of the newly assembled collagen fibrils around the lacunae. An example of this is shown by the two lacunae in Supplementary Movie 3 that are indicated by arrowheads. Figure 2C shows still frames of the same two forming lacunae from the boxed region indicated in Figure 2A. Note that between 5 and 5.4 days, the holes indicated by arrowheads are formed within the collagen fibril network. This occurs by cell(s) physically pushing the collagen fibrils outward to form an initial hole, followed by assembly of more collagen around the periphery of the lacunae and compaction of the collagen into a dense fibril network. During the time period from d4 to d8, there was still continuous cell movement and fibril stretching as well as some condensation of fibrils occurring in certain regions, especially between days 6 and 8 (see Supplementary Movie 3).

Supplementary Movie 4 shows the time period from d8 to d16.7 (also see still frames in Figures 2A,B). Please note from this movie that the mature collagen matrix at d8 appears relatively stable, with little motion compared to earlier time points. The first sign of mineral deposition can be seen in the DIC movie panel and still frames at around day 9, with rapid mineral deposition occurring between days 10 and 12. After mineralization, as would be predicted, there was very little movement of collagen within the mineralized nodule area. However, there was continued fibril motion in areas that were not mineralized. Figure 2B shows still frame images of GFP-collagen, DIC and a merged GFP-collagen/DIC image at day 16.7. The outline of the mineralized nodule is indicated in yellow and it is clear that mineral was deposited only in areas containing bright, dense, GFP-collagen that appears distinct from the collagen in the non-mineralized areas.

**FIGURE 3:**
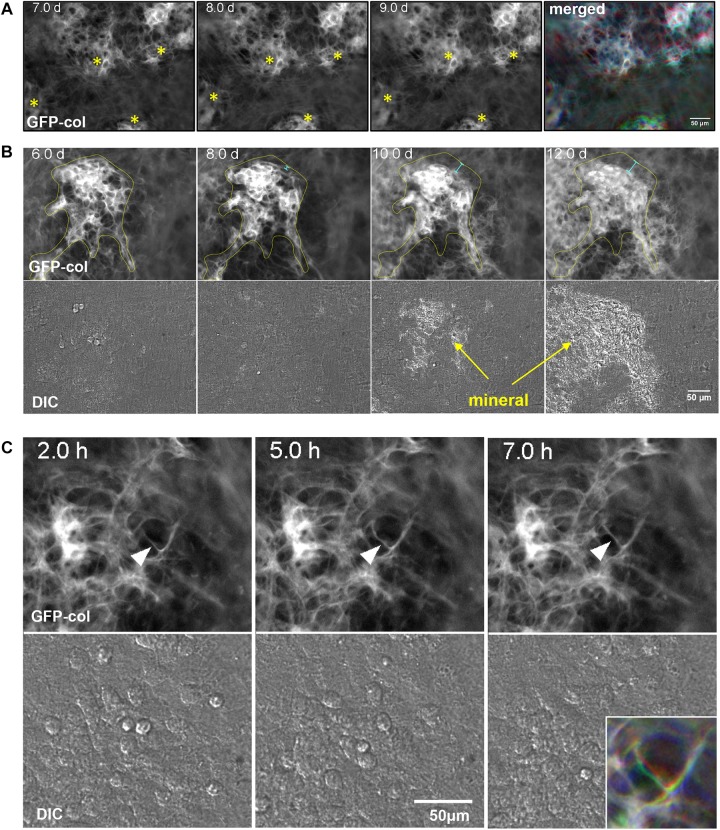
Dynamic events during collagen assembly. **(A)** Still frames from timelapse imaging of bone nodules forming in long-term osteogenic calvarial cell cultures. Note the nodules marked with the asterisks that move relative to each other. This is best appreciated by viewing Supplementary Movie 5. The RGB color image on the right shows the 7, 8, and 9 day images merged into the red, green, and blue channels respectively. **(B)** Still frames from timelapse imaging of a bone nodule mineralizing. The collagen in the osteogenic nodule is outlined in yellow at day 6 and the same outline is superimposed on the images at days 8, 10, and 12. The light blue distance bars indicate compaction of the collagen relative to the original contours of the nodule. This is best appreciated by viewing Supplementary Movie 7. **(C)** Still frames from a timelapse movie illustrating local motions of an individual fibril marked with an arrowhead. The fibril starts out straight at 2 h and is deformed by local cell motion at 5 h before becoming straightened again at 7 h. These local fibril dynamics are best appreciated by viewing Supplementary Movie 8, where you can see this and other fibrils repeatedly being stretched and distorted by local cell motions. The colored inset image in the lower right shows the same fibril from the 2, 5, and 7 h images merged into the red, green, or blue channels respectively. In all panels, bar = 50 μm.

In many cases, when mineralized bone nodules were forming, global (tissue-level) motions were observed in which the forming nodules moved relative to each other, suggesting coordinated movement of sheets of cells between the nodules. This is best appreciated by viewing Supplementary Movie 5 and is illustrated by the still images in Figure 3A. When viewing Supplementary Movie 5, note that the bone nodule foci marked by asterisks in Figure 3A show motions relative to each other and that the one at the bottom of the image field moves up and down relative to the other nodules. This is further illustrated by the merged color image in Figure 3A in which still frames from 7, 8, and 9 days are pseudocolored in red, green and blue, respectively, and merged. Areas that appear white indicate no change, but areas that are red, green or blue indicate motions of the forming nodules relative to each other.

Intravital imaging in the calvaria of GFP-collagen expressing mice provided analogous observations whereby the collagen in the mineralizing bone matrix appeared structurally distinct from the collagen fibers in the sutures. Global motions of the mineralizing bone fronts on either side of the suture relative to each other were observed, apparently due to contraction of collagen in the sutures, presumably due to underlying cell and tissue-level motions (see Supplementary Movie 6). Interestingly, the structural appearance of bone collagen in the calvarial bone in the live mice resembled that in the mature *in vitro* bone nodules after mineralization (compare Supplementary Movie 6 with the GFP-collagen image in Figure 2B). However, our intravital imaging studies in mice have so far been limited to timescales of up to 8 h and this was not sufficiently long to see significant new collagen deposition.

As well as motions of bone nodules relative to each other, compaction/condensation of collagen within mineralizing foci was frequently observed prior to the onset of mineralization. This is best appreciated by viewing Supplementary Movie 7 in which compaction of collagen in the nodule can be observed at around days 7–8, preceding mineral deposition, which starts at around day 9. This is further illustrated by the still images in Figure 3B in which the forming bone nodule is outlined in yellow at day 6. The day 6 outline is superimposed on the 8, 10, and 12 day images, showing that the contours of the nodule are reduced relative to day 6, as indicated by the light blue distance bars. DIC images show mineral deposition at 10 and 12 day.

In addition to global motions of fibrils and condensation of fibril networks prior to mineral deposition, localized motions of individual fibrils were also frequently observed. An example of this is shown in Supplementary Movie 8, in which the fibril marked by the arrowhead is repeatedly distorted relative to those around it, presumably due to local cell motion/protrusive activity. Still images in Figure 3C illustrate motions of the fibril in Supplementary Movie 8. The motion of this fibril is best appreciated by the merged color image shown in the lower right inset, in which still frames from 2, 5, and 7 h are pseudocolored blue, green and red, respectively and merged. Areas that appear white indicate no change, but areas that are red, green or blue indicate motions of the fibril.

### Dmp1-Cre/tdTomato Cell Dynamics During Collagen Assembly

**FIGURE 4:**
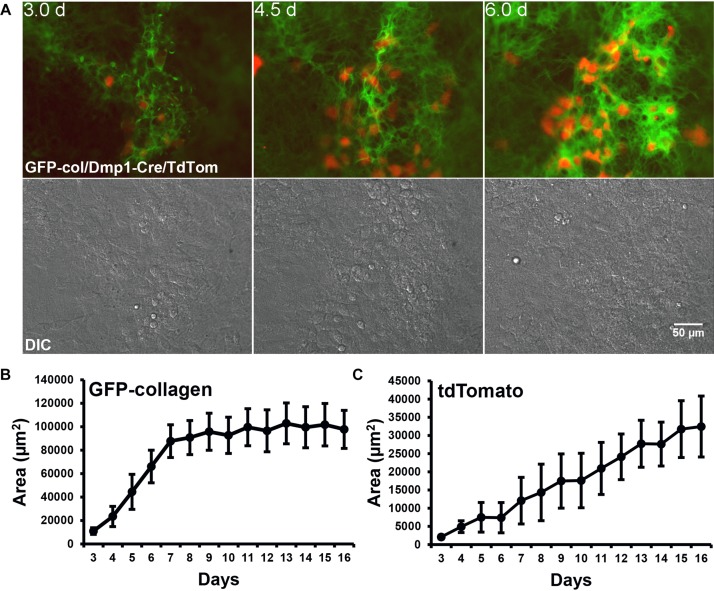
Dmp1-Cre/tdTomato cell dynamics during collagen assembly. **(A)** Still frames from timelapse dual imaging of GFP-collagen (green) and Dmp1-Cre/tdTomato (red) in primary calvarial osteoblast cultures between days 3 and 6 (upper panels). DIC images of the cell cultures are shown in the lower panels. Bar = 50 μm. To appreciate the dynamic process of collagen assembly together with tdTomato cell dynamics please view Supplementary Movie 9. **(B,C)** Quantitative analysis of the area of GFP-collagen **(B)** and area of tdTomato-positive cells **(C)** in long-term osteogenic calvarial cell cultures over days 3–17. Data are mean ± SEM from *n* = 4 independent movies.

Next, to visualize how osteocyte differentiation is dynamically integrated with bone collagen assembly, two-color movies of GFP-collagen were examined together with Dmp1-Cre/tdTomato to visualize preosteocytes/osteocytes. Interestingly, the tdTomato reporter turned on almost exclusively in the same foci of concentrated GFP-collagen fibers that demarcate where mineralized nodules will form (Figure 4A and Supplementary Movie 9) with very little expression of tdTomato in regions between bone nodule foci. As can be seen in Supplementary Movie 9, the tdTomato turns on first in cells that are motile, but as the forming bone nodule matures, many of them become embedded in lacunae within the collagen matrix. Quantitation of the area of GFP-collagen and tdTomato fluorescence showed that over the 2 week osteogenic differentiation time course, the majority of collagen assembled within the first 7 days, whereas the number of Dmp1-Cre/tdTomato cells continuously increased throughout the time course (Figures 4B,C). Interestingly, a more detailed analysis with measurements taken at every time point revealed small spikes in the area of GFP-collagen fluorescence that appeared to correspond to media changes every 48 h (Supplementary Figure S1A). This probably reflects initial rapid assembly and intracellular accumulation of collagen upon changing the media and adding fresh ascorbate. A similar detailed analysis showed a stepwise increase in tdTomato fluorescence area that also seemed to correspond with changes in media every 48 h (Supplementary Figure S1B). These kinetics are missed in most standard types of experiments that do not have the increased temporal resolution provided by time lapse imaging with images acquired every 30 min.

**FIGURE 5:**
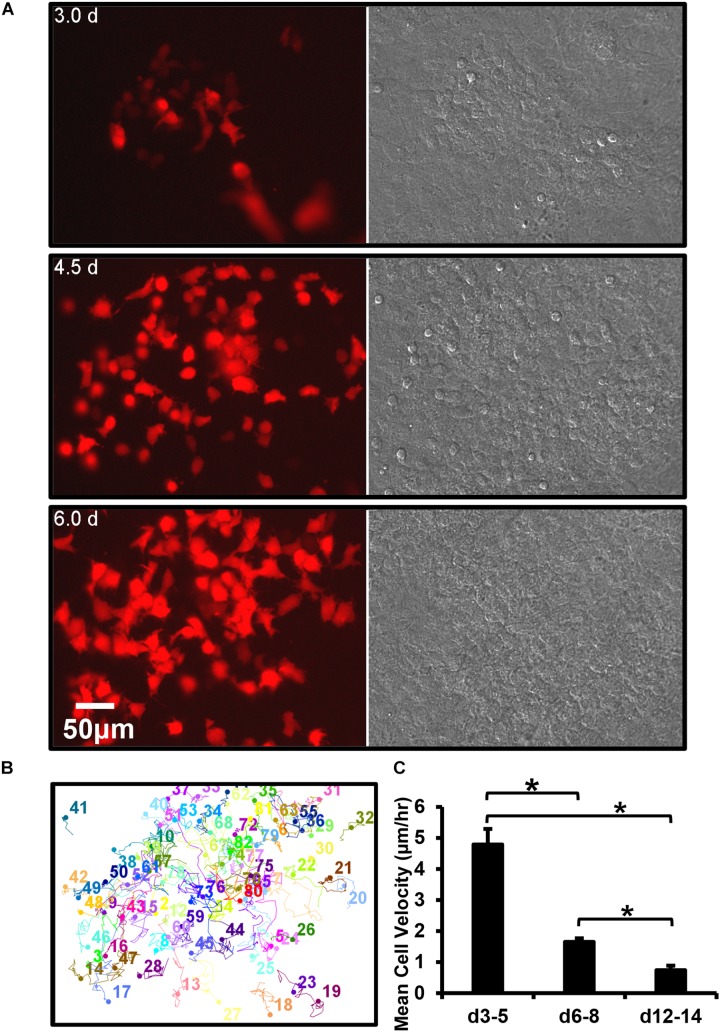
Dmp1-Cre/tdTomato reporter switches on in a post-mitotic cell that is initially motile and then embeds. **(A)** Still frames from a timelapse movie showing the dramatic increase in tdTomato-positive cells over time in osteogenic calvarial cell cultures from Dmp1-Cre/tdTomato transgenic mice, bar = 50 μm. The tdTomato-positive cells arise exclusively from new cells switching on tdTomato rather than from mitotic division of cells already expressing tdTomato, which is best appreciated by viewing Supplementary Movie 10. **(B)** Motion trajectory plots for individual cells within the movie field. **(C)** Graph showing the average velocities of tdTomato-positive cells over 3–5, 6–8, and 12–14 days. Data are mean ± SEM from *n* = 9 independent movies for d3–5 and *n* = 10 for d6–8 and d12–14. ^∗^*p* < 0.05 (ANOVA/Student Newman Keul’s).

The dramatic increase in tdTomato-positive cells that occurred throughout the differentiation process (Figures 4A,C, 5A) could either be due to mitosis of tdTomato-positive cells or due to individual cells switching on tdTomato expression during differentiation. Quantitative analysis from 5 independent movies (total of 103 cells analyzed) showed that 100% of these tdTomato-positive cells arose by cells switching on tdTomato and no instances were recorded of cells arising through mitosis of a tdTomato-positive cell. This is illustrated in Supplementary Movie 10, where numerous cells can be observed to turn on tdTomato expression without originating from a cell division. Supplementary Movie 10 is formatted so that after playing once through, it plays back in reverse. The cell marked with the arrowhead in the reverse movie segment is an example of a cell that can be tracked back to its origin to confirm that it arose by switching on tdTomato rather than from a cell division. These data show that the Dmp1-Cre/tdTomato cells are post-mitotic. These post-mitotic tdTomato-positive cells make up a relatively small fraction of the entire population in the calvarial cell cultures and their numbers increase over time as more mineralized nodules are formed. Within the mineralizing nodule regions themselves, quantitative analysis indicated that the tdTomato positive cells occupy 28–30% of the nodule area (data not shown). In a typical differentiated primary calvarial cell culture, the mineralized bone nodule area occupies about 25–40% of the culture plate area. Therefore, the overall percentage of tdTomato-positive cells is estimated to be around 7–12% of the total population.

Motility tracking analysis was performed on tdTomato-positive cells from long-term time lapse movie stacks (Figure 5B). This showed that in early (d3–5) cultures the cells moved freely in and around the bone forming nodules prior to mineralization. Quantitative analysis showed that they had a mean velocity of 4.8 μm/h (Figure 5C). At this early time, only a few cells were embedded in collagen, constraining their motion. However, in more mature (d6–8), cultures the mean velocity was significantly reduced to 1.7 μm/h and in mineralizing (d12–14) cultures it was further reduced to 0.7 μm/h, as many cells became immobilized in the mineralized nodule (Figure 5C).

### Osteocyte Embedding Behavior Prior to Mineralization

**FIGURE 6:**
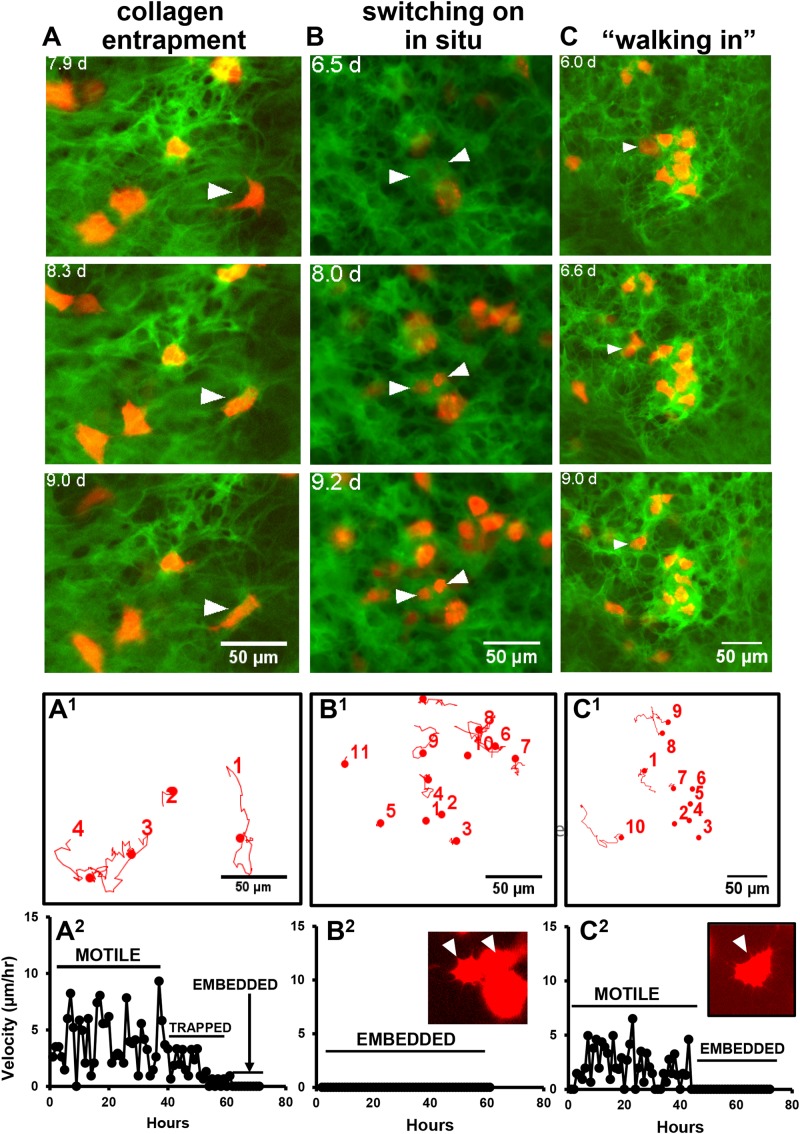
Multiple mechanisms for osteocyte embedding. **(A–C)** Still frames from timelapse movies that illustrate the different mechanisms of embedding of tdTomato-positive cells in osteogenic calvarial cell cultures from GFP-collagen/Dmp1-Cre/tdTomato transgenic mice. **(A)** shows a tdTomato-positive cell (arrowhead) becoming embedded via collagen entrapment. This cell initially shows motile activity until around 8 days, when a network of collagen fibers is assembled around the cell, thereby immobilizing it. This is best visualized dynamically by viewing Supplementary Movie 11; **(B)** shows two cells (arrowheads) that switch on tdTomato expression *in situ* while already located within a lacuna. This is best visualized dynamically by viewing Supplementary Movie 12; **(C)** shows a tdTomato-positive cell (arrowhead) that shows motile activity and “walks into” an already formed lacuna in the collagen matrix. The contours of the cell then conform to the contours of the lacuna. This is best visualized dynamically by viewing Supplementary Movie 13. **(A^1^–C^1^)** Show motion trajectory plots for the cells in movies **(A–C)**, respectively. **(A^2^–C^2^)** Show the motility profiles for the featured cells in **(A–C)**, respectively, that exemplify the different embedding behaviors. The inset images show that the morphology of the embedded cells in movies **(B,C)** at the end of the movie resemble the dendritic morphology of osteocytes. In all panels, bar = 50 μm.

Dual imaging of GFP-collagen and Dmp1-Cre/tdTomato provided novel insight into how osteocytes may embed into their lacunae. Interestingly, three distinct mechanisms for embedding were observed. In some instances, previously motile tdTomato-positive cells became trapped and immobilized in collagen fibril networks that were newly assembled around the cell. An example of this is shown in Figure 6A and Supplementary Movie 11. Note the cell marked with the arrowhead that is freely motile at the start of the movie. By around 8 days, the motility of the cell is arrested as it is trapped in a network of new collagen fibers assembled around it. In other cases, cells appeared to switch on tdTomato expression *in situ* within an already formed presumptive lacuna in the collagen matrix. An example of this is shown in Figure 6B and Supplementary Movie 12. Note the two cells marked with arrowheads that turn on tdTomato expression when they are already located within a presumptive lacuna, suggesting that the cells were already present in the lacunae prior to differentiating to express Dmp1. A third mechanism was observed in which tdTomato-positive cells appeared to “walk” into an already formed hole/lacuna in the collagen network. Once immobilized, the cell then adopted a morphology conforming to the contours of the lacuna. An example of this is shown in Figure 6C and Supplementary Movie 13. Motion trajectory plots of the cells in each movie are shown in Figure 6 in the corresponding panels A^1^, B^1^, and C^1^. The cell motility profiles of the featured cells showing “collagen entrapment,” “switching on *in situ*” and “walking into lacunae” behaviors are shown in Figure 6 in the corresponding panels A^2^, B^2^, and C^2^. As expected, the cell motility plots paralleled the behavior of the cells observed in the timelapse movies, with the “collagen entrapped” cell (A^2^) initially showing motile behavior, followed by a reduction in motility after entrapment and a further reduction to zero once the cell is fully embedded. The “switching on *in situ*” cell (B^2^) showed no motile behavior at all, consistent with the observation that it appeared to differentiate from a cell that was already located within a lacuna. The inset image shows that one of these two cells also adopts a dendritic osteocyte morphology. The third type of cell that “walks into a lacuna” (C^2^) shows a period of motility before it “walks” into the lacuna and then shows no further motile behavior after it is embedded. In another variation on the “walking into a lacuna” embedding style, several cells were observed that appeared to move into and out of more than one presumptive lacunae before finally embedding. An example is shown in Supplementary Figure S2 and Supplementary Movie 14. Here, two lacunae are marked by the yellow arrowheads and the moving cell is marked by the white arrowhead. The motion trajectory plot of this cell shows a period of motility, then a period of inactivity while the cell is temporarily located in lacuna L1. This is followed by another period of motility while the cell moves to the second lacuna and finally the motility is reduced to zero when the cell embeds in lacuna L2. Supplementary Figures S3–S5 show 10 examples of individual cell motility profiles for each category of embedding cell, including the “collagen entrapped” (Supplementary Figure S3), “switching on *in situ*” (Supplementary Figure S4) and “walking into a lacuna” (Supplementary Figure S5) embedding types as well as a graph showing the mean velocities of the 10 cells of each type. Although there is some variation in the profiles amongst individual cells, the cells within each category show similar motility profiles that parallel their embedding behaviors. Comparisons of the mean velocities of these cells over the first 40 h of tracking showed that the “collagen entrapped” cells had a significantly higher velocity (4.621 μm/h ± 0.418) compared to either the “switching on *in situ*” (0.014 μm/h ± 0.005) or “walking into a lacuna” cells (2.599 μm/h ± 0.0.499) (*p* < 0.05, ANOVA/Tukeys).

**FIGURE 7:**
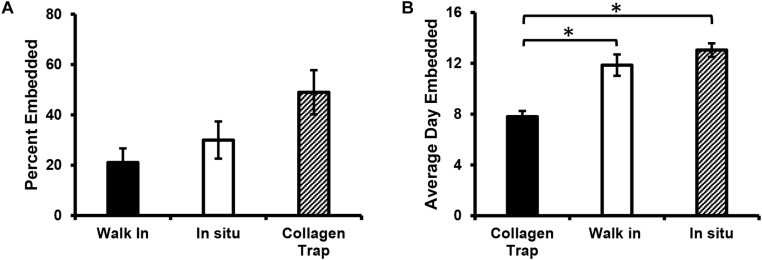
Mechanism of osteocyte embedding may be dependent on the maturation of the bone nodule. **(A)** Quantitation of the percentage of Dmp1-Cre/tdTomato-positive cells that embed via the three different mechanisms identified, including collagen entrapment, switching on tdTomato *in situ* and “walking” into an already formed collagen lacuna. **(B)** Graph showing the average day on which embedding occurred for cells in the three embedding categories. Data in **(A,B)** are mean ± SEM from *n* = 4 independent movies (86 individual embedding cells analyzed). **(B)** ^∗^*p* < 0.05, ANOVA/Tukeys.

Quantitation of the overall percentages of cells embedding by each type of mechanism showed that the majority of the cells (49%) embedded by collagen entrapment, with 30% turning on tdTomato expression *in situ* and 21% embedding by “walking into” an already formed lacuna (see Figure 7A). When the average day of embedding was determined for each of these categories, it was found that the collagen entrapment mechanism tended to be used by cells that embedded significantly earlier (average around 8 days), compared to the other two mechanisms, which tended to be used by cells that embedded later (average around 12–13 days) (see Figure 7B).

## Discussion

In this study, long-term timelapse imaging was performed in mineralizing bone cell cultures, which has provided novel insight into the dynamic process of collagen assembly and mineralization and has revealed three independent mechanisms by which osteocytes can embed within bone matrix. This timelapse imaging was performed using primary calvarial cell cultures from transgenic mice expressing GFP-collagen together with a tdTomato lineage reporter for differentiation toward the osteocyte phenotype. Long-term live imaging of collagen deposition in this osteogenic culture model showed that, while collagen is assembled throughout the cell layer, mineral deposition is restricted to regions demarcated by foci of concentrated GFP-collagen bundles that appear structurally distinct from the surrounding collagen. These foci are associated with cell condensations that demarcate where the bone nodule will form. The assembly of collagen within these foci was highly dynamic, with global (tissue level) motions of the collagen, including an outward expansion of collagen that appeared to be mediated by coordinated movement of cells outwards from the center of the nodule, as well as motions of the forming bone nodules relative to each other, suggesting coordinated movement of sheets of cells between the nodules. In addition, there were local motions of individual fibrils that were stretched/distorted, presumably by local cell motions or protrusive activity. These observations on collagen assembly dynamics show parallels with the work of Rongish and co-workers, who examined fibrillin and fibronectin fibril dynamics in early avian embryos (Filla et al., 2004; Czirok et al., 2006; Aleksandrova et al., 2012). Their work identified two distinct types of motion of ECM filaments, one in which the filaments are moved by large-scale tissue motions due to folding of tissue layers during embryonic morphogenesis and a second type whereby the motion of individual filaments is driven by motility/protrusive activity of neighboring cells. Snapping and recoil of fibronectin fibrils by local cell-mediated forces has also been demonstrated using live cell imaging (Ohashi et al., 1999; Sivakumar et al., 2006; Davidson et al., 2008). We have also recently reported on the generation of GFP*topaz* and mCherry-tagged type I collagen fusion protein constructs that were stably transfected into MLO-A5 osteoblast-like cells and fibronectin-null mouse embryonic fibroblasts (Lu et al., 2018). Timelapse imaging in these cell models over timeframes of up to 2 days revealed the dynamic nature of type I collagen assembly and showed that collagen assembly was dependent on and temporally integrated with fibronectin assembly (Lu et al., 2018). These studies also showed that the cells were able to physically reshape their assembled collagen fiber networks by pushing collagen outwards to form holes that may represent early lacunae. Similar physical reshaping of the collagen matrix was observed in the current study and the formation of future osteocyte lacunae appeared to be mediated by cells pushing collagen fibrils outwards from the center of the forming lacuna, accompanied by deposition of more collagen and compaction of the collagen fibril networks around the lacuna. A further overall condensation of the collagen fibrils in the forming bone nodule was observed that preceded mineral deposition.

A key issue to consider is how the dynamic collagen assembly events observed in the current study in an osteogenic cell culture model can be extrapolated to assembly and mineralization of collagen *in vivo*. In the long bones, which contain mostly lamellar bone, collagen is deposited in a highly organized manner by osteoblasts aligned on a mineralization front, and the collagen fibers are predominantly oriented longitudinally. The osteogenic primary cell culture model used in the current studies is derived from calvarial bone, which is formed developmentally by intramembranous ossification. Therefore, this model is probably more relevant to intramembranous and/or woven bone formation, where collagen is deposited rapidly, with a less organized and more random orientation. In the current study, intravital imaging was performed in GFP-collagen transgenic mice and it was observed that the structural and organizational appearance of collagen in calvarial bone in the growth regions adjacent to the sutures is very similar to its organization in mineralizing calvarial cell cultures and that the collagen in the mineralizing bone matrix appeared structurally distinct from the collagen fibers in the sutures. Similar to data from the *in vitro* osteogenesis model, global motions of the mineralizing bone fronts on either side of the suture relative to each other were observed, apparently due to cell-mediated contraction of collagen in the sutures. However, in our experience the collagen assembly process is relatively slow and our intravital imaging in mice has so far been limited to timescales of up to 8 h, which is not sufficiently long to visualize significant assembly of new collagen. Intravital imaging using transgenic mice co-expressing GFP-collagen and Dmp1-Cre/tdTomato also did not enable us to visualize osteocyte embedding events, due to the limited timescales for intravital imaging (data not shown). In contrast, the *in vitro* osteogenic cell culture model enables tracking of multiple embedding events that take place over the extended timescales of 7–14 days that can be imaged using this model system.

In the current study, time lapse imaging in osteogenic calvarial cell cultures showed that the Dmp1-Cre/tdTomato reporter turned on in the same foci of concentrated GFP-collagen bundles that progress to form mineralized bone nodules, with very little expression in regions between bone nodules. The number of Dmp1-Cre/tdTomato-positive cells continuously increased throughout the 2 week timecourse, whereas the majority of collagen was assembled by day 7. In early forming bone nodules, the tdTomato reporter was expressed initially in a motile cell type. Timelapse imaging allowed observation of the fate of these Dmp1-Cre/tdTomato-positive cells over a long timecourse and this revealed that cells which turned on tdTomato expression almost invariably became embedded within “lacunae” in the collagen matrix, which later mineralized. The Dmp1-Cre transgene therefore appears to mark a cell type that is already committed to becoming a future osteocyte. This observation is important, as when initially developed, the Dmp1-Cre deleter strain was thought to specifically target gene deletion to osteocytes (Lu et al., 2007). Later studies showed apparent off target expression in bone in a subpopulation of cells on the bone surface that by their location would be viewed as osteoblasts (Kalajzic et al., 2013; Dallas et al., 2018). However, the current studies suggest that these cells represent a late osteoblast/preosteocyte that is already committed to becoming an embedded osteocyte. The fact that the tdTomato-positive cells arose exclusively by new cells turning on tdTomato expression rather than from mitosis of tdTomato-positive cells further supports this, by demonstrating that the Dmp1-Cre/tdTomato-positive cells are a post-mitotic, committed cell.

Although we have virtually never observed the tdTomato-positive cells to divide in this osteogenesis culture model, it was reported recently that Dmp1-expressing osteocyte-like cells have the capacity to dedifferentiate into osteoblasts and proliferate. These observations were made using an *in vitro* assay in which the cells migrate out from bone chips onto a plastic culture surface (Torreggiani et al., 2013). In considering this data together with the findings of the current study, a likely explanation is that the Dmp1-expressing cell will retain its post-mitotic state as long as it remains surrounded by bone extracellular matrix, which provides signaling cues to the cell to maintain its differentiated state. The dedifferentiation of osteocyte-like cells most likely occurs only if the cell is removed from its microenvironment and is separated from these environmental cues, for example by migrating out onto plastic. It remains unclear whether such dedifferentiation of osteocytes can occur *in vivo*.

Long term time lapse imaging in the current study has shed new light on the unresolved question of how osteoblasts embed to become osteocytes. A widely accepted theory proposes that embedding is a passive process in which specific surface osteoblasts slow down their production of extracellular matrix and then become “buried alive” in the osteoid produced by adjacent cells (Palumbo et al., 1990; Nefussi et al., 1991; Franz-Odendaal et al., 2006). A competing theory proposes that osteocyte embedding is an active, invasive process, involving proteolytic degradation of the extracellular matrix to excavate the osteocyte lacuna and canaliculi (Zhao et al., 2000; Holmbeck et al., 2005). Our data suggest that there may be more than one mechanism for osteocytes to become embedded in bone extracellular matrix. In some cases, a motile Dmp1-Cre/tdTomato-positive cell, presumably representing a late osteoblast/preosteocyte, became entrapped in a network of collagen fibers that was newly assembled around the cell. The probable source of this newly assembled collagen appears to be the adjacent cells, as GFP-collagen was not observed inside tdTomato-positive cells. Therefore, this seems to agree with theories of osteoblasts being “buried alive” in the extracellular matrix produced by neighboring cells (Palumbo et al., 1990; Nefussi et al., 1991; Franz-Odendaal et al., 2006). A second intriguing mechanism observed was one in which a motile tdTomato-positive late osteoblast/preosteocyte cell “walked in” to an already formed collagen lacuna in the developing nodule. This was followed by arrest of cell motility and the cell morphology adopting a shape conforming to the boundaries of the lacuna. There were also variations on this theme in which cells were seen to move from one lacuna to another before finally settling into a lacuna. Since the current study was limited to imaging the collagen matrix and Dmp1-Cre\tdTomato-positive cells, it is unclear whether another cell that is tdTomato-negative would have to vacate the lacuna to allow the tdTomato-positive cell to occupy it. Future studies using multiplexed imaging with an osteoblast reporter in addition to imaging collagen and Dmp1-Cre/tdTomato-positive osteocytes are needed to address this question. The third mechanism for osteocyte embedding involved cells that were already located within a lacuna differentiating *in situ* to turn on expression of Dmp1-Cre/tdTomato. Therefore, cells that fell into this category showed no motility after switching on tdTomato.

Which of these three mechanisms is used appears to be time-sensitive and/or dependent on the maturation state of the forming bone nodule, with the collagen entrapment mechanism being favored at earlier stages of bone nodule formation, when more of the cells were motile, rapid collagen deposition was occurring and the collagen fibrillar networks showed more motion. The other two mechanisms were more predominant at later stages of bone nodule formation when the collagen fibril networks were more mature and more rigid, collagen assembly had plateaued and mineral deposition had begun. At these later stages, the presumptive osteocyte lacunae were also well demarcated. In extrapolating these observations to bone formation *in vivo*, it seems likely that different embedding mechanisms could operate depending on the maturation state of the forming bone matrix and/or the rapidity with which the bone is being formed. For example, in early stages of bone formation and in the rapid type of bone formation that produces woven bone, when copious amounts of collagen are quickly being assembled, entrapment of osteocytes within newly assembled collagen matrix may be the predominant mechanism. In contrast, in formation of lamellar bone or later stages of woven bone formation when collagen assembly is slower, the other two mechanisms may predominate. Regardless of the mechanism, osteocyte embedding appears to be a systematic rather than a random process, as osteocytes have a regular spacing within the bone matrix when viewed in three dimensions, with approximately equal distances between neighboring osteocytes.

In summary, we present for the first time, long-term timelapse dual imaging of collagen assembly dynamics and osteocyte embedding using an *in vitro* osteogenic cell culture model. These data have revealed the highly dynamic nature of collagen assembly and maturation and provided new insight into the dynamic process of osteocyte differentiation and embedding. Rather than a single mechanism for osteocyte embedding, the data suggest multiple mechanisms for osteocyte entrapment in collagen matrix that may be time and matrix maturation dependent.

## Data Availability

The raw data supporting the conclusions of this manuscript will be made available by the authors, without undue reservation, to qualified researcher.

## Ethics Statement

The animal study was reviewed and approved by UMKC Animal Care and Use Committee.

## Author Contributions

SD, LS, and YL designed the study and interpreted the data. LS, LT-L, YX, and ER collected and analyzed the data. LS and SD drafted the manuscript. LT-L, YX, YL, and ER revised and approved the manuscript. SD took responsibility for the integrity of the data.

## Conflict of Interest Statement

The authors declare that the research was conducted in the absence of any commercial or financial relationships that could be construed as a potential conflict of interest.
