# Supplementary material for: Collagen Dynamics During the Process of Osteocyte Embedding and Mineralization
Source: Front Cell Dev Biol. 2019 Sep 18;7:178. doi: 10.3389/fcell.2019.00178 (PMC6759523; doi:10.3389/fcell.2019.00178)
Supplement: Supplementary file 15 [file Data_Sheet_1.PDF]

Supplementary Material

1. Supplementary Figures

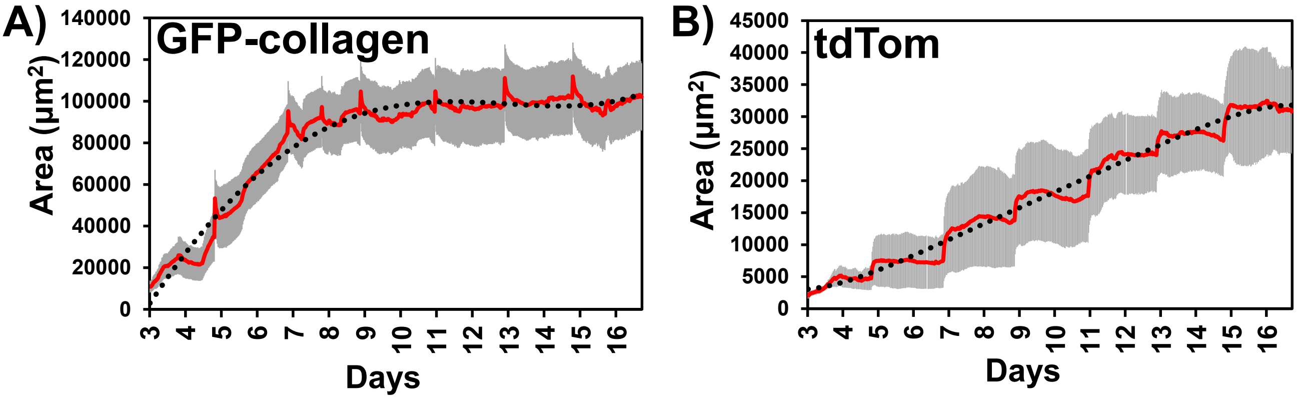

Figure S1: Detailed Timecourse of GFP-Collagen Deposition And tdTomato Expression in Long Term Osteogenic Calvarial Cell Cultures. Quantitation of **A)** Area of GFP-collagen and **B)** Area of tdTomato-positive cells from timelapse movies in osteogenic calvarial cell cultures. Images were acquired every 30 minutes from days 3-17, providing a higher resolution of the data compared to standard experiments without timelapse imaging. The red trace shows the measured data, the dashed line shows a polynomial curve fit to the measured data and the grey lines show error bars for each individual time point. Note that there is a small spike in GFP-collagen deposition at intervals approximately 48h apart and that there is a step up in tdTomato-positive cells that occurs with a periodicity of about 48h. Data are mean ± SEM from n=4 independent movies.

A)

B)

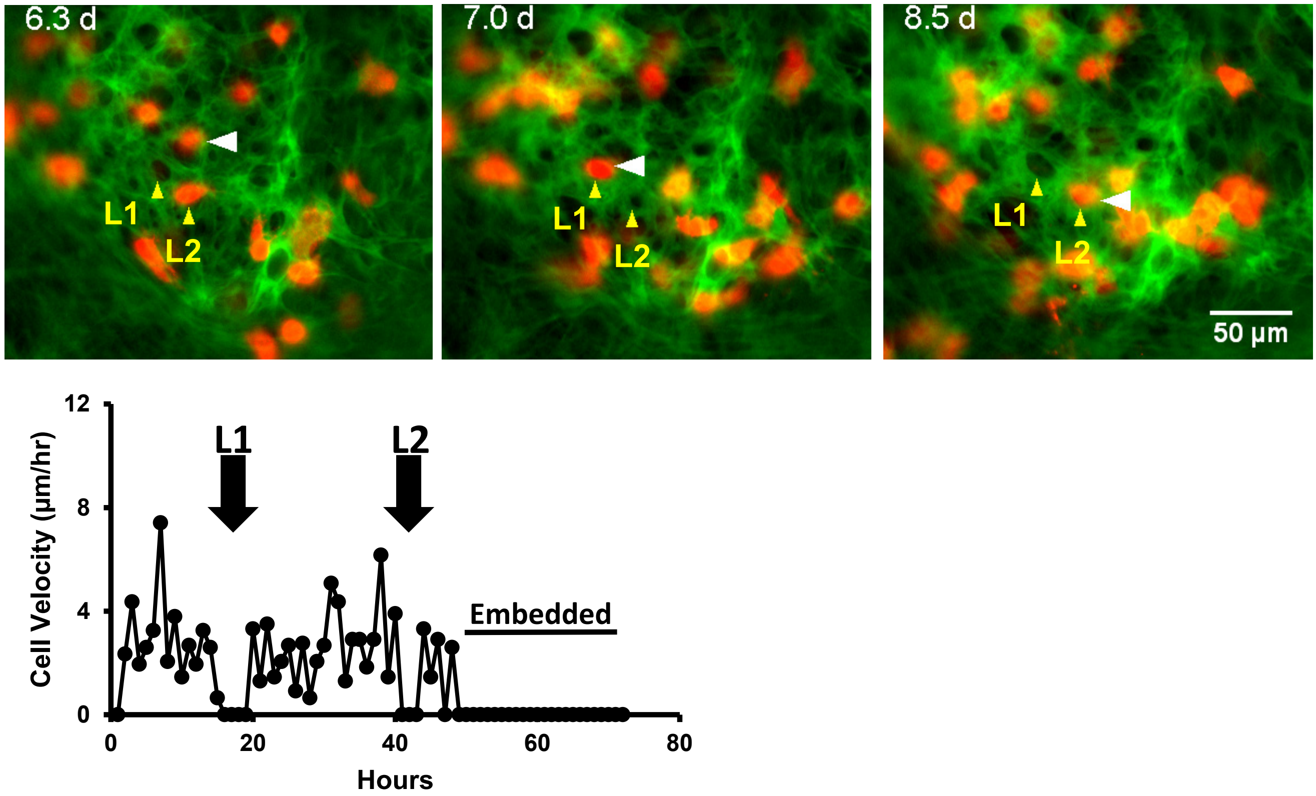

Figure S2: Variation on tdTomato-positive Cell "Walking into" a Preformed Lacuna. A) Still frames from a timelapse move in an osteogenic calvarial cell culture from transgenic mice expressing GFP-collagen/Dmp1-Cre/tdTomato. Note the cell marked with the white arrowhead. This cell moves first into one lacuna (L1) and then into a second lacuna (L2). The cell embeds in the second lacuna and adopts a shape that conforms to the boundary of the lacuna. The dynamics of this process are best appreciated by viewing ***supplementary movie*** ***14***. **B)** Motility profile for the cell marked with the white arrowhead. The cell shows periods of motile behavior in between periods of inactivity when the cell was located first in lacuna 1 (L1) and then in lacuna 2, where it becomes immobilized (L2). Bar = 50µm.

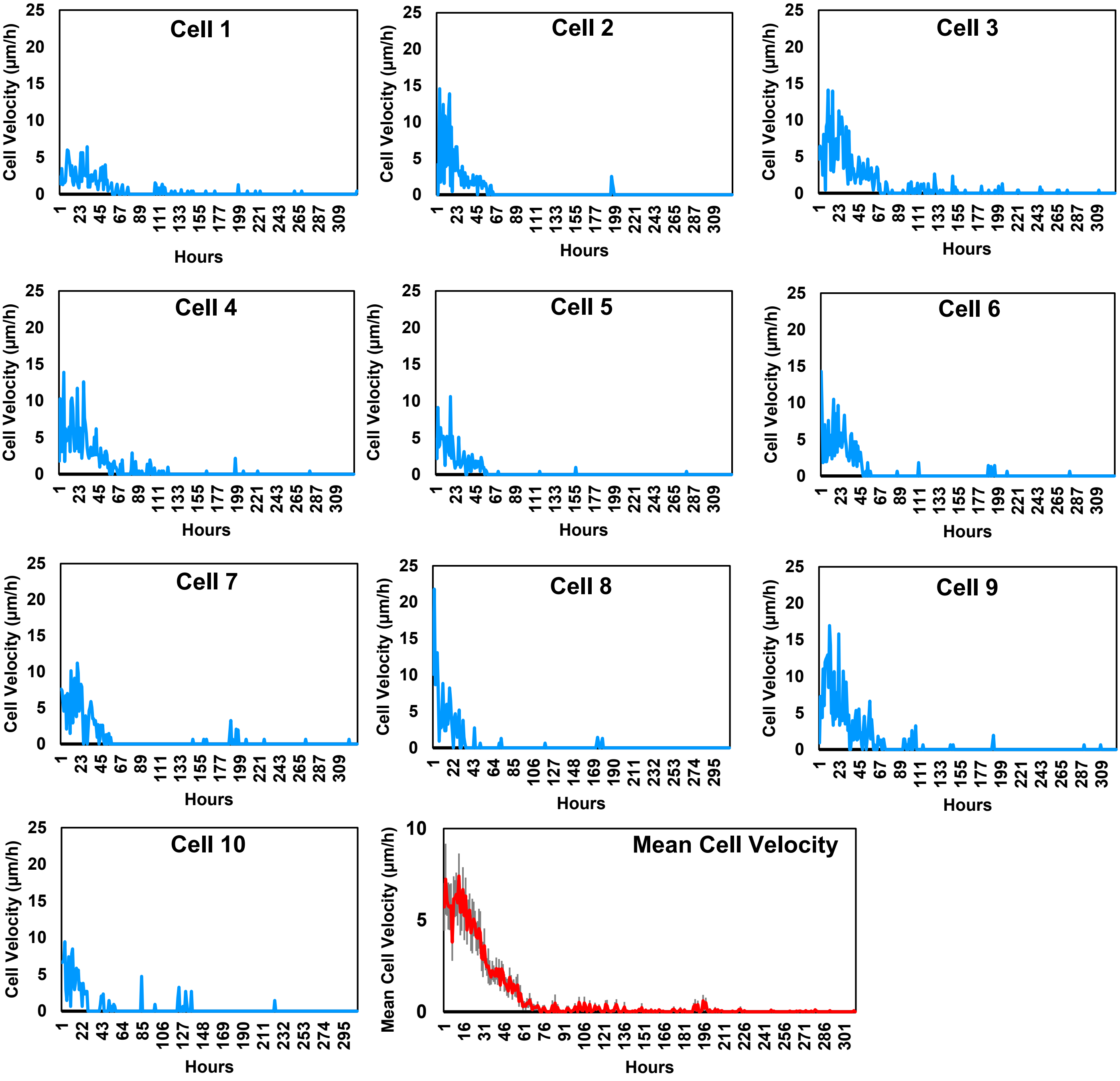

Figure S3: Individual Motility Profiles for 10 Examples of Cells that Embed via the Collagen Entrapment Mechanism. Motility plots are shown for 10 individual Dmp1-Cre/tdTomato cells that show embedding by collagen entrapment. A graph of the average cell velocity is also shown for these 10 cells (red trace), with error bars shown in grey (mean ± SEM).

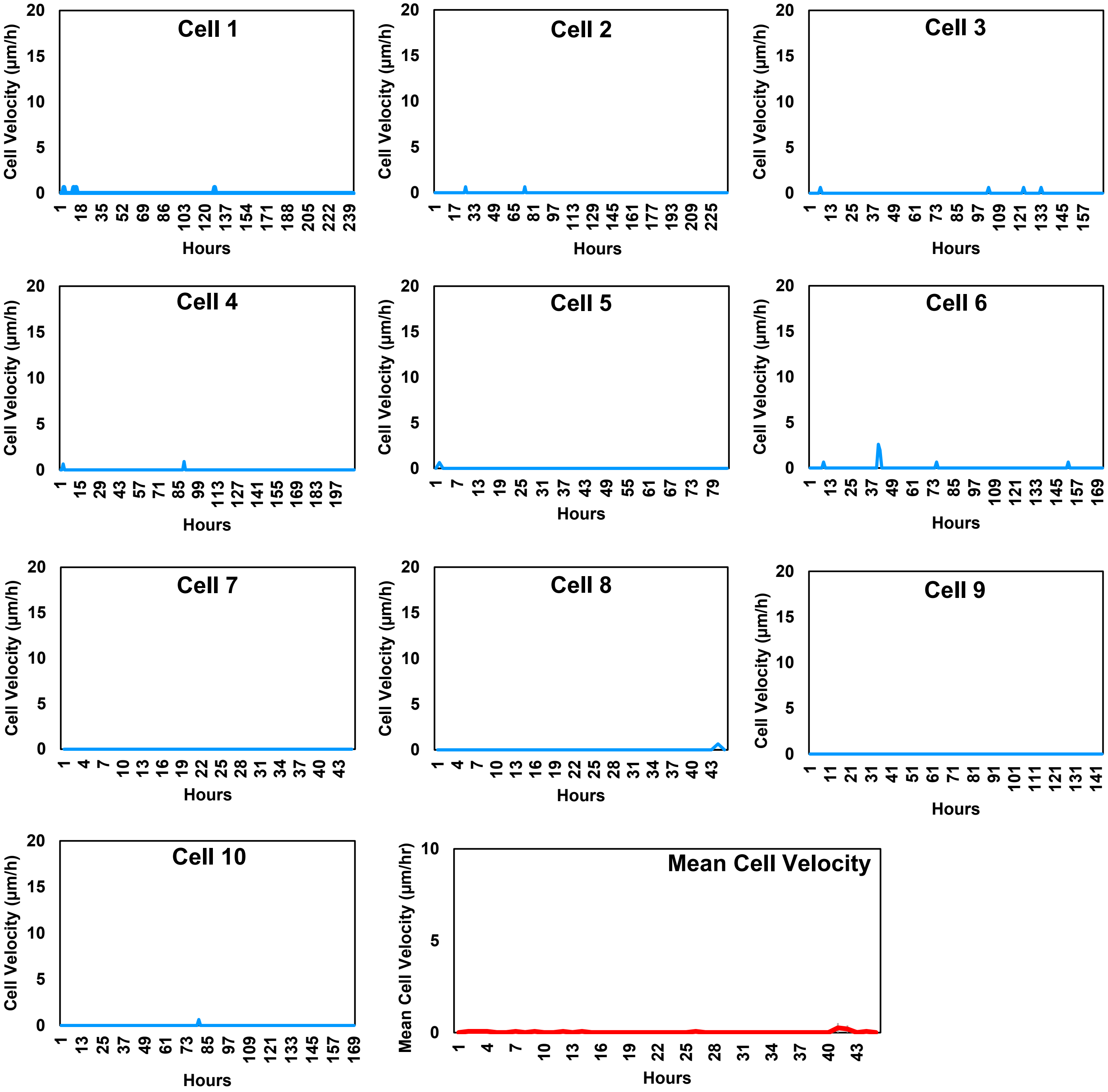

Figure S4: Individual Motility Profiles for 10 Examples of Cells that Embed via the "Switching on *in Situ*" Mechanism. Motility plots are shown for 10 individual Dmp1-Cre/tdTomato cells that show embedding by *in situ* switching on of tdTomato.A graph of the average cell velocity is also shown for these 10 cells (red trace), with error bars shown in grey (mean ± SEM).

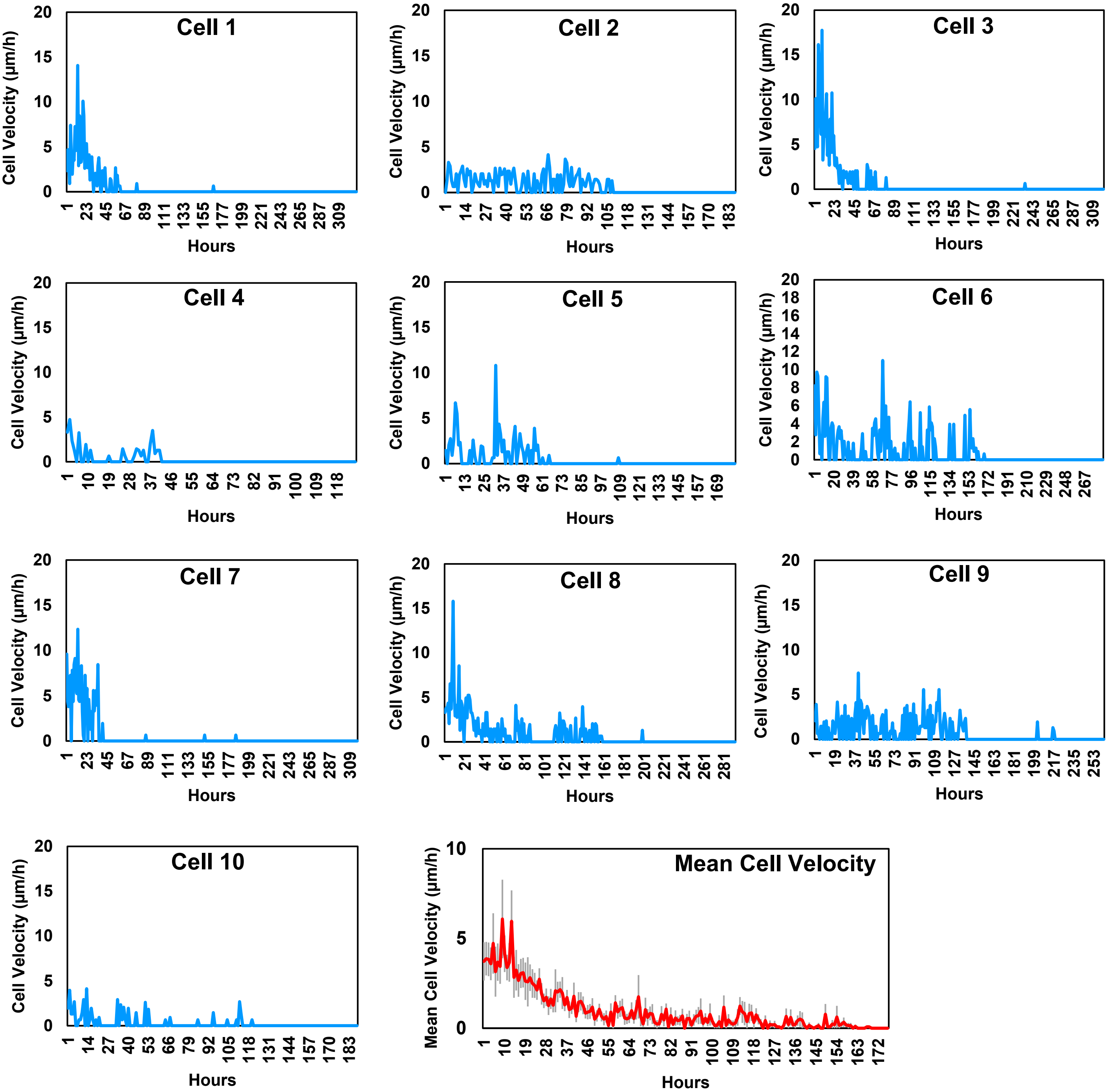

Figure S5: Individual Motility Profiles for 10 Examples of Cells that Embed via the "Walking into a Lacuna" Mechanism. Motility plots are shown for 10 individual Dmp1-Cre/tdTomato cells that show embedding by "walking into" a preformed lacuna. A graph of the average cell velocity is also shown for these cells (red trace), with error bars shown in grey (mean ± SEM)

2. Legends for Supplementary Movies

Supplementary Movie 1: Long-term Live Imaging of GFP-Collagen Assembly in Osteogenic Calvarial Cell Cultures. [LEFT PANEL: GFP-collagen; RIGHT PANEL: DIC]. Timelapse movie of long-term imaging of GFP-collagen deposition in a mineralizing osteogenic calvarial cell culture. This movie depicts the entire period from day 3, when the forming bone nodule is visible as a condensation of cells in the DIC image through to 16.7 days, when the bone nodule is mineralized. Note that the cell condensations are associated with a bright focal area of GFP-collagen that appears structurally distinct from the fainter collagen in the surrounding cell layer. Both the cells and collagen fibrils show considerable motion until later time points in the movie when mineral deposition on the collagen prevents further motion. This movie is played at a frame rate of 40fps to depict the entire culture period, but movies 2, 3 and 4 show shorter segments of the movie at different stages to further illustrate the dynamic events during collagen assembly. Bar = 40µm. [gamma adjustment 0.5 applied to GFP-collagen movie stack].

Supplementary Movie 2: Early Stages of Bone Nodule Formation and GFP-Collagen Assembly in Osteogenic Calvarial Cell Cultures. [LEFT PANEL: GFP-collagen; RIGHT PANEL: DIC]. Timelapse movie showing a shorter segment from Movie 1 from day 3-4. The movie is played at a slower frame rate of 10fps to illustrate early events in collagen assembly during early nodule formation. The initial formation of a future bone nodule is seen in the DIC images as a condensation of cells in a focal area where the cells became rounded/multilayered. Note that these cells also show increased membrane ruffling compared to surrounding cells, visible as bright hair-like features on the cell surface. Although faint GFP-collagen fibers are seen in the surrounding cell layer, the GFP-collagen is brighter and more concentrated where the cells are condensing. The collagen in the developing bone nodule undergoes expansion/stretching and appears to be pushed outwards from the forming nodule via coordinated movement of cells outwards from the center of the nodule. There is also extensive assembly of new collagen in the forming nodule and the adjacent cell layer and further condensation of cells to increase the size of the nodule. Note that the cells are constantly in motion, which exerts forces on the fibrils resulting in stretching/small deformations during their assembly and expansion. Bar = 40µm. [gamma adjustment 0.5 applied to GFP-collagen movie stack].

Supplementary Movie 3: Intermediate Stage of Bone Nodule Formation and GFP-Collagen Assembly in Osteogenic Calvarial Cell Cultures. [LEFT PANEL: GFP-collagen; RIGHT PANEL: DIC]. Timelapse movie showing a segment from Movie 1 covering the period from d4 to d8 (frame rate 10fps). Note that over d4-d8, the collagen in the forming nodule continues to expand outwards and brighter and thicker GFP-collagen fibril networks are assembled. The collagen in the forming bone nodule appears structurally distinct from the surrounding collagen and by day 7 has a honeycomb-like appearance, with several hole-like structures that will later become osteocyte lacunae. The yellow arrows illustrate two lacunae that form by cells initially pushing the collagen outwards to form a hole in the fibril network. This is followed by new collagen assembly and condensation around the periphery of the forming lacunae. Over d4 to d8, there is also continuous cell movement and fibril stretching. Bar = 40µm. [gamma adjustment 0.5 applied to GFP-collagen movie stack].

Supplementary Movie 4: Late Mineralizing Stage of Bone Nodule Formation and GFP-Collagen Assembly in Osteogenic Calvarial Cell Cultures. [LEFT PANEL: GFP-collagen; RIGHT PANEL: DIC]. Timelapse movie showing a segment from Movie 1 covering the period from d8 to d16.7 (frame

rate 20fps). Note that there is not much more collagen deposition over this time period and the mature collagen matrix appears relatively stable, with less fibril motion compared to earlier time points. Mineral starts to be deposited at around day 9 (indicated by yellow arrows and text). The mineral is deposited only in the nodule areas containing bright, condensed collagen fibril networks and not on collagen in the adjacent cell layer. Bar = 40µm. [gamma adjustment 0.5 applied to GFP-collagen movie stack].

Supplementary Movie 5: Global (Tissue Level) Motions Between Forming Bone Nodules in Osteogenic Calvarial Cell Cultures. [LEFT PANEL: GFP-collagen; RIGHT PANEL: DIC]. Timelapse movie in a long-term osteogenic calvarial cell culture from day 5 to day 13.2. This movie illustrates global (tissue level) motions occurring whereby the forming bone nodules move relative to each other (a frame rate of 80fps is used to illustrate this). Extensive local motions of fibrils can also be seen. Bar = 50µm. [gamma adjustment 0.5 applied to GFP-collagen movie stack].

Supplementary Movie 6: Intravital Imaging of GFP-Collagen in Calvarial Bone in a 15 Day Old GFP-Collagen Transgenic Mouse. Timelapse movie of intravital imaging of the calvarium of a 15 day old GFP-collagen transgenic mouse (6.25h duration, frame rate 7fps). Note the similar structural appearance of the bone collagen compared to collagen in osteogenic calvarial cell cultures after bone nodule mineralization (supplementary movies 1 and 4 and fig, 2b in main manuscript). Also note the structurally distinct appearance of the collagen in the sutures compared to the bone. Global (tissue level) motions of the mineralizing bone fronts on either side of the suture relative to each other were observed, apparently due to contraction of the collagen in the suture. Within the 6.25h timescale of this movie, significant deposition of new collagen is not seen. Bar = 30µm.

Supplementary Movie 7: Collagen Compaction Prior to Mineralization in Osteogenic Calvarial Cell Cultures. [LEFT PANEL: GFP-collagen; RIGHT PANEL: DIC]. Timelapse movie in a long-term osteogenic calvarial cell culture from day 6.3 to day 11.5 (frame rate 50fps). This movie illustrates contraction of the collagen fibril network that occurs in mineralizing nodules over days 6.3 to 9.5 prior to mineral deposition that starts on d9.5. Bar = 50µm. [gamma adjustment 0.5 applied to GFP-collagen movie stack].

Supplementary Movie 8: Local Collagen Fibril Motions in Osteogenic Calvarial Cell Cultures. [LEFT PANEL: GFP-collagen; RIGHT PANEL: DIC].Timelapse movie in a long-term osteogenic calvarial cell culture showing the time period from 50h to 66h after initiation of imaging at day 3 (frame rate 10fps). The arrowheads highlight two collagen filaments that show local stretching/distortion due to the underlying cell motion. Several other examples of fibrils showing local motions can be observed in the movie. Bar = 50µm. [gamma adjustment 0.5 applied to GFP-collagen movie stack].

Supplementary Movie 9: GFP-Collagen Assembly Dynamics and Dmp1-Cre/TdTomato Cell Dynamics in Osteogenic Calvarial Cell Cultures. [LEFT PANEL: GFP-collagen (green) and Dmp1-Cre/tdTomato (red); RIGHT PANEL: DIC]. Timelapse movie in a long-term osteogenic calvarial cell culture from day 3 to day 6 showing collagen assembly and osteocyte differentiation monitored using GFP-collagen and the Dmp1-Cre/tdTomato reporter, respectively (frame rate 20fps). Note that induction of tdTomato expression is localized to the same foci of concentrated GFP-collagen fibers that demarcate where mineralized nodules will form. There is very little expression of tdTomato in regions between bone nodule foci. The number of tdTomato-positive cells increases throughout the movie. Cells expressing tdTomato appear motile initially, but later several become embedded in lacunae in the

collagen network, while others remain motile. Bar = 50µm. [gamma adjustment 0.5 applied to GFP-collagen movie stack].

Supplementary Movie 10: Dmp1-Cre/tdTomato Reporter Initially Turns on in a Motile Cell Population that is Post-Mitotic. [LEFT PANEL: Dmp1-Cre/tdTomato; RIGHT PANEL: DIC]. Timelapse movie in a long-term osteogenic calvarial cell culture from day 3 to day 6.2 (frame rate 15 fps). This movie is configured to play forwards from day 3 to day 6.2 and then play in reverse from day 6.2 back to day 3. Note that the tdTomato reporter turns on initially in cells that are motile and that by 6.2 days many cells appear to have become immobilized (presumably embedded), while others remain motile. The increase in tdTomato-positive cells over time occurs due to cells switching on tdTomato rather than via mitotic division of tdTomato-positive cells. The tdTomato-positive cells therefore appear to be post-mitotic. For the reverse segment of the movie, the cell marked with the arrowhead provides an example of a cell that can be tracked back to confirm that it arose by switching on tdTomato expression rather than through mitotic division. Bar = 50µm. [gamma adjustment 0.6 applied to GFP-collagen movie stack].

Supplementary Movie 11: Osteocyte Embedding Via Collagen Entrapment. [GFP-collagen (green), Dmp1-Cre/tdTomato (red)]. Timelapse movie in a long-term osteogenic calvarial cell culture from day 6 to day 9 (frame rate 20 fps). Note the tdTomato-positive cell indicated with the arrowhead that initially shows motile behavior. By around 8.1 days, the cell starts to become immobilized as a network of collagen fibrils is newly assembled around it, thereby entrapping it. [gamma adjustment 0.5 applied to GFP-collagen movie stack].

Supplementary Movie 12: Osteocytes Switching on Dmp1-Cre/tdTomato Expression In Situ Within a Lacuna. [GFP-collagen (green), Dmp1-Cre/tdTomato (red)]. Timelapse movie in a long-term osteogenic calvarial cell culture from day 6.3 to day 9.2 (frame rate 20 fps). Note the two lacunae within the collagen network at the beginning of the movie indicated by the arrowheads. By around day 6.4, two cells within these lacunae turn on expression of the tdTomato reporter and remain immotile within the lacunae. [gamma adjustment 0.5 applied to GFP-collagen movie stack].

Supplementary Movie 13: Osteocytes Embedding Via "Walking in" to a Lacuna. [GFP-collagen (green), Dmp1-Cre/tdTomato (red)]. Timelapse movie in a long-term osteogenic calvarial cell culture from day 6 to day 9 (frame rate 20 fps). Note the tdTomato-positive cell marked by the arrowhead. This cell is initially motile and at around 7.8 days it appears to move into a lacuna in the collagen network. It then becomes immobilized and the contour of the cell adopts the contour of the lacuna. [gamma adjustment 0.5 applied to GFP-collagen movie stack].

Supplementary Movie 14: Variation on "Walking in" to a Lacuna Embedding Mechanism. [GFP-collagen (green), Dmp1-Cre/tdTomato (red)]. Timelapse movie in a long-term osteogenic calvarial cell culture from day 6.3 to day 11.3 (frame rate 20 fps). Note the tdTomato-positive cell marked by the arrowhead. This cell is initially motile and around 7 days it appears to move into a lacuna marked as L1 in the collagen network. It then moves out of this lacuna and later moves into a second lacuna, marked L2 by around 8 days. The cell then becomes immobilized in the L2 lacuna and the contour of the cell adopts the contour of the lacuna. [gamma adjustment 0.5 applied to GFP-collagen movie stack].
